# The Association of Urinary Sodium Excretion with Glaucoma and Related Traits in a Large United Kingdom Population

**DOI:** 10.1016/j.ogla.2024.04.010

**Published:** 2024-05-08

**Authors:** Kelsey V. Stuart, Mahantesh I. Biradar, Robert N. Luben, Neeraj Dhaun, Siegfried K. Wagner, Alasdair N. Warwick, Zihan Sun, Kian M. Madjedi, Louis R. Pasquale, Janey L. Wiggs, Jae H. Kang, Marleen A.H. Lentjes, Hugues Aschard, Jihye Kim, Paul J. Foster, Anthony P. Khawaja

**Affiliations:** 1 NIHR Biomedical Research Centre, Moorfields Eye Hospital NHS Foundation Trust and UCL Institute of Ophthalmology, London, UK.; 2 MRC Epidemiology Unit, University of Cambridge, Cambridge, UK.; 3 Edinburgh Kidney, University/BHF Centre of Research Excellence, The Queen’s Medical Research Institute, University of Edinburgh, Edinburgh, UK.; 4 UCL Institute of Cardiovascular Science, University College London, London, UK.; 5 Department of Ophthalmology, University of Calgary, Calgary, Alberta, Canada.; 6 Department of Ophthalmology, Icahn School of Medicine at Mount Sinai, New York, New York.; 7 Department of Ophthalmology, Massachusetts Eye and Ear, Harvard Medical School, Boston, Massachusetts.; 8 Department of Medicine, Brigham and Women’s Hospital, Harvard Medical School, Boston, Massachusetts.; 9 School of Medical Sciences, Örebro University, Örebro, Sweden.; 10 Department of Computational Biology, Institut Pasteur, Université Paris Cité, Paris, France.; 11 Department of Epidemiology, Harvard T.H. Chan School of Public Health, Boston, Massachusetts.

**Keywords:** Dietary salt, Gene-environment interaction, Glaucoma, Intraocular pressure, Urinary sodium

## Abstract

**Purpose:**

Excessive dietary sodium intake has known adverse effects on intravascular fluid volume and systemic blood pressure, which may influence intraocular pressure (IOP) and glaucoma risk. This study aimed to assess the association of urinary sodium excretion, a biomarker of dietary intake, with glaucoma and related traits, and determine whether this relationship is modified by genetic susceptibility to disease.

**Design:**

Cross-sectional observational and gene-environment interaction analyses in the population-based UK Biobank study.

**Participants:**

Up to 103 634 individuals (mean age: 57 years; 51% women) with complete urinary, ocular, and covariable data.

**Methods:**

Urine sodium:creatinine ratio (UNa:Cr; mmol:mmol) was calculated from a midstream urine sample. Ocular parameters were measured as part of a comprehensive eye examination, and glaucoma case ascertainment was through a combination of self-report and linked national hospital records. Genetic susceptibility to glaucoma was calculated based on a glaucoma polygenic risk score comprising 2673 common genetic variants. Multivariable linear and logistic regression, adjusted for key sociodemographic, medical, anthropometric, and lifestyle factors, were used to model associations and gene-environment interactions.

**Main Outcome Measures:**

Corneal-compensated IOP, OCT derived macular retinal nerve fiber layer and ganglion cell-inner plexiform layer (GCIPL) thickness, and prevalent glaucoma.

**Results:**

In maximally adjusted regression models, a 1 standard deviation increase in UNa:Cr was associated with higher IOP (0.14 mmHg; 95% confidence interval [CI], 0.12-0.17; *P* < 0.001) and greater prevalence of glaucoma (odds ratio, 1.11; 95% CI, 1.07-1.14; *P* < 0.001) but not macular retinal nerve fiber layer or ganglion cell-inner plexiform layer thickness. Compared with those with UNa:Cr in the lowest quintile, those in the highest quintile had significantly higher IOP (0.45 mmHg; 95% CI, 0.36-0.53, *P* < 0.001) and prevalence of glaucoma (odds ratio, 1.30; 95% CI, 1.17-1.45; *P* < 0.001). Stronger associations with glaucoma (*P* interaction = 0.001) were noted in participants with a higher glaucoma polygenic risk score.

**Conclusions:**

Urinary sodium excretion, a biomarker of dietary intake, may represent an important modifiable risk factor for glaucoma, especially in individuals at high underlying genetic risk. These findings warrant further investigation because they may have important clinical and public health implications.

**Financial Disclosure(s):**

Proprietary or commercial disclosure may be found in the Footnotes and Disclosures at the end of this article.

Glaucoma, a highly heritable disease characterized by progressive optic neuropathy, is the leading cause of irreversible blindness worldwide.^1,2^ Global prevalence is estimated at 76 million, with projections rising to 112 million by 2040.^3^ Elevated intraocular pressure (IOP) represents the only known modifiable risk factor for the disease, and all current glaucoma therapies work by lowering IOP.^2^ In addition to IOP-mediated mechanical stress, it has also been postulated that vascular and neurodegenerative mechanisms may underlie the pathophysiology of glaucoma.^2^

The etiology of glaucoma is complex and multifactorial, with numerous genetic and environmental determinants thought to play a role.^4^ Recent advances in glaucoma genetic discovery and polygenic risk score (PRS) development have now made it possible to identify high-risk individuals before the clinical onset of disease, and the identification of environmental factors that could potentially modify genetic risk is a particular research priority.^4,5^

Excessive dietary sodium intake is an important cardiovascular risk factor, estimated to cause 5 million deaths per annum worldwide, through an association with elevated blood pressure.^6^ This relationship is thought to be mediated primarily through alterations in intravascular fluid volume, adverse vascular remodeling, and autonomic nervous dysfunction.^7^ Although systemic hypertension has previously been implicated as a potential risk factor for glaucoma, the association between dietary sodium intake and glaucoma is less clear.^8^ Self-reported dietary salt consumption was recently reported to be adversely associated with prevalent primary open-angle glaucoma (POAG) but only among hypertensive medication users, in the Thessaloniki Eye Study (TES).^9^

The estimation of sodium intake based on dietary analysis is difficult and the validity generally low.^10,11^ Because the majority of dietary sodium is excreted via the kidneys, urinary sodium excretion represents an objective and reliable biomarker of dietary intake.^6,12^ The purpose of this study was therefore to assess the association of urinary sodium excretion with glaucoma and related traits, including IOP and OCT derived measures of inner retinal thickness, on a population level, because a better understanding of these relationships may have important clinical and public health implications.

## Methods

### Study Population

The UK Biobank is a large population-based cohort study and data resource of ~500 000 individuals aged 37 to 73 years at recruitment (2006–2010). Participants were recruited through National Health Service registers and invited to attend one of 22 assessment centers across the United Kingdom where extensive phenotypic information and biological samples were collected.^13,14^ After providing electronic informed consent, participants completed an in-depth touchscreen questionnaire (detailing sociodemographic information, life-course exposures, and medical history) and an array of physical and cognitive measurements. Blood, urine, and saliva specimens were also collected and used to generate a wealth of genetic, proteomic, and metabolomic data.^15^ Multiple repeat and supplementary assessments, including an eye and vision substudy (2009–2010), have been conducted in participant subsets to augment the baseline data.^16^ Additional health-related outcomes are available through linkage with nationwide medical records and registries. Detailed descriptions, including the overall study protocol and individual test procedures, are available online (https://www.ukbiobank.ac.uk).

### Ethical Approval

The UK Biobank was approved by the National Health Service North West Multicentre Research Ethics Committee (06/MRE08/65) and the National Information Governance Board for Health and Social Care. This research was conducted under UK Biobank application number 36741 and conformed to the tenets of the Declaration of Helsinki. All participants provided electronic informed consent.

### Assessment of Urinary Sodium Excretion

From 2006 to 2010, approximately 485 000 UK Biobank participants provided a midstream urine sample as part of the baseline assessment.^15^ Specimens were packaged and refrigerated according to protocol before being transported overnight by a dedicated commercial courier to a central laboratory. Samples were then processed and 9 mL urine aliquots stored in ultralow temperature archives. A predefined panel of biomarkers, including sodium (coefficient of variation [CV], 1%), potassium (CV, 1%), and creatinine (CV, 2%), were assayed using a single Beckman Coulter AU5400 clinical chemistry analyzer (Beckman Coulter UK, Ltd) using the manufacturer’s reagents and calibrators. The Beckman Coulter AU5400 series uses a potentiometric measurement for the determination of sodium and potassium concentrations, and a photometric measurement for the determination of creatinine concentration. Each assay was validated against the manufacture’s performance information and linearity experiments determined the reportable range. For each assay, the observed reportable range covered the manufacturer’s analytical range (sodium, 10-400 mmol/L; potassium, 2-200 mmol/L; creatinine, 88-44 200 mmol/L). To account for variable urine concentration, we calculated the urine sodium:creatinine ratio (UNa:Cr; mmol:mmol) from these specimens. In a steady state, renal excretion of creatinine remains relatively constant, and the urinary creatinine concentration therefore provides a measure of the state of dilution or concentration of the urine. This approach is widely used to estimate 24-hour excretion of sodium and other analytes, such as albumin and catecholamines, from spot urine samples.^17^ The urine sodium:creatinine ratio in the top and bottom percentiles of the distribution were excluded. Full details of the urine assays and quality control information for the urinary biomarker data are available online (https://biobank.ndph.ox.ac.uk/ukb/ukb/docs/urine_assay.pdf).

In addition, a subset of approximately 70 000 participants completed a 24-hour dietary assessment (Oxford WebQ questionnaire) as part of their baseline assessment.^18^ Estimated nutrient intake, including dietary sodium (mg), has been calculated for these participants using food composition data from the United Kingdom Nutrient Databank and was used to assess the relationship between urinary sodium excretion and reported dietary intake.^19^

### Assessment of Glaucoma-Related Outcome Measures and Glaucoma Case Ascertainment

The UK Biobank eye and vision substudy was introduced as an enhancement in 2009 to 2010 and generated additional ophthalmic data for a subset of participants.^16^

Intraocular pressure measurements in both eyes of approximately 115 000 participants were taken using an Ocular Response Analyzer non-contact pneumotonometer (Reichert Corp).^16^ Participants reporting an eye infection or eye surgery within the previous 4 weeks did not undergo IOP assessment. Individual-level IOP values were calculated as the mean of available right and left eye values, and extreme IOP values in the top and bottom 0.5 percentiles were excluded. For this analysis, we used corneal-compensated IOP, a measure derived from a linear combination of inward and outward applanation tensions that is least influenced by corneal biomechanical properties.^20^ We excluded participants with a history of glaucoma surgery or laser therapy, corneal graft or refractive surgery, or visually significant ocular trauma because these participants are likely to have IOP that has been altered from physiological levels (these exclusions were not applied to the analyses of OCT parameters or glaucoma status). We imputed pretreatment IOP values for participants using ocular hypotensive agents by dividing the measured IOP by 0.7, based on the mean IOP reduction achieved by medication, as previously described.^21,22^

Macular spectral domain OCT imaging using a Topcon 3D OCT-1000 Mark II (Topcon Corp) was performed on both eyes of approximately 65 000 participants.^16^ The image handling, segmentation and quality control protocols have been described previously.^23^ Briefly, scans were performed in a dark room without pupil dilation using the 3D 6 × 6 mm^2^ macular volume scan mode (512 A-scans per B-scan; 128 horizontal B-scans in a raster pattern). Version 1.6.1.1 of the Topcon Advanced Boundary Segmentation algorithm was used to delineate the inner and outer retinal surfaces.^24^ We excluded scans with an image quality score (signal strength) less than 45. Additionally, several segmentation indicators were calculated that also identified poor scan quality or segmentation failures; we excluded the poorest 20% of images for each of these indicators. For this analysis, we used macular retinal nerve fiber layer (mRNFL) and ganglion cell-inner plexiform layer (GCIPL) thicknesses, both averaged across the Macula 6 grid, as these measures have been shown to be useful glaucoma-related biomarkers.^25,26^ We calculated individual level OCT values as the mean of all available right and left eye measurements.

From 2006 to 2010, the touchscreen questionnaire administered to approximately 175 000 participants included a question on physician-diagnosed eye disorders. Participants were considered cases if they reported a diagnosis of glaucoma, or previous surgical or laser treatment for glaucoma, in either eye. We also included any participant carrying an International Classification of Diseases (ICD) code for glaucoma (ICD ninth revision: 365.* [excluding 365.0]; ICD 10th revision: H40.* [excluding H40.0] and H42.*) in their linked hospital records at any point prior to, and up to 1 year after, the baseline assessment. We excluded cases who were diagnosed prior to 30 years of age, and controls who reported using ocular hypotensive medication or carrying an ICD code for glaucoma suspect (ICD ninth revision: 365.0; ICD 10th revision: H40.0).

### Genotyping and PRS

Genetic data for approximately 490 000 UK Biobank participants were generated using 2 closely related genotyping platforms. The Affymetrix UK BiLEVE Axiom Array returned genotypes at 807 411 markers for approximately 50 000 participants, while the Affymetrix UK Biobank Axiom Array provided genotypes at 825 925 markers for the remaining approximately 440 000 participants.^27^ Quality control and imputation were performed jointly for these 2 platforms, as previously described.^14^ Imputation (genotypic determination based on inference and not by direct typing) was based on the UK10K and Haplotype Reference Consortium reference panels. To assess whether observed exposure-outcome associations were modified by genetic factors (gene-environment interaction), we constructed a PRS based on 2673 independent single nucleotide polymorphisms associated with glaucoma (at *P* ≤ 0.001) from a recent multitrait genome-wide association study meta-analysis of European participants.^5^ Glaucoma is a complex polygenic disease, and we considered the PRS to be a better representation of genetic risk in glaucoma than any individual or limited set of variants. We used the effect estimates from the original genome-wide association study to generate a glaucoma PRS for each participant using a standard weighted sum of individual single nucleotide polymorphisms:

$$\sum_{i=1}^{2673}{\hat{\beta }}_{i}*SNP_{i}$$

where ${\hat{\beta }}_{i}$ is the estimated effect size of $SNP_{i}$ on glaucoma. The PRS was standardized with a mean of 0 and a standard deviation (SD) of 1 for analyses.^5^

### Assessment of Covariables

We considered a range of sociodemographic, medical, anthropometric, and lifestyle factors in our analyses based on previously reported risk factors for glaucoma, associations with IOP, or determinants of urinary sodium excretion. All covariables used in this analysis were ascertained at the time of the baseline assessment and on the same day as the urine collection and ophthalmic assessment. These included: age (years), sex (women, men), self-reported ethnicity (White, Asian, Black, and Mixed/other), Townsend deprivation index (a measure of material deprivation based on an individual’s residential postcode; a higher index score indicates greater relative poverty), height (cm), weight (kg), systolic blood pressure (SBP; mmHg; calculated as the mean of 2 measurements), glycated hemoglobin (mmol/mol), total cholesterol (mmol/L), smoking status (never, current, former), alcohol intake (g/day),^28^ physical activity (metabolic equivalent of task-minutes/week; a measure of energy expenditure based on an adapted version of the validated International Physical Activity Questionnaire),^29^ assessment season (Summer, Autumn, Winter, Spring), time of urine collection (morning, 06h00–12h00; afternoon, 12h00–18h00; evening, 18h00–00h00), and urinary potassium concentration (mmol/L). Full details of these variables, including protocols, equipment, procedures, and descriptive statistics are available online (https://www.ukbiobank.ac.uk).

### Statistical Analysis

Baseline participant characteristics were summarized as mean (SD) for continuous variables, and frequency (proportion) for categorical variables. The linear-by-linear and Cochrane-Armitage tests were used to assess trends across UNa:Cr quintiles, as appropriate. To assess the main associations between urinary sodium excretion and the various glaucoma-related outcomes, we used multivariable linear (for IOP, mRNFL thickness, and GCIPL thickness) and logistic (for glaucoma) regression models adjusted for the covariables described above. Given the strong causal relationship between dietary salt intake and hypertension and to assess whether any associations may be mediated through blood pressure, we considered multivariable regression models both without, and with, adjustment for SBP. All other covariables were considered potential confounders and were included in both sets of regression models. Urinary sodium excretion was analyzed as both a continuous (standardized UNa:Cr) and categorical (quintiles of UNa:Cr) variable. Trends across quintiles were examined by testing the median value of each group. To assess whether any associations were modified by the glaucoma PRS, we tested the significance of a multiplicative interaction term between the standardized UNa:Cr and standardized PRS in the final multivariable models using the Wald test. Gene-environment interaction analyses were restricted to participants of European ancestry based on principal components analysis. All analyses were performed using Stata (Version 17.0. StataCorp LLC. 2021).

### Sensitivity Analyses

Given that urinary sodium excretion may be influenced by antihypertensive medication use or renal impairment, we performed stratified analyses by self-reported use of any blood pressure medication and estimated glomerular filtration rate (eGFR) categories. The eGFR calculations were based on the revised 2021 Chronic Kidney Disease Epidemiology Collaboration formulae.^30^ We also adjusted final models for both eGFR and urine microalbumin concentration (mg/L) to further account for possible confounding by renal impairment. We performed sex-stratified analyses because women have been shown to have a greater susceptibility to salt-sensitive hypertension than men, and additionally adjusted all models for systemic beta-blocker use and caffeine intake, based on previously reported associations.^31-33^ Lastly, to account for potential misclassification bias, we examined associations with several alternative glaucoma case definitions: (i) self-reported glaucoma only, (ii) ICD-coded glaucoma only (excluding H40.0, glaucoma suspects), (iii) ICD-coded POAG only, and (iv) history of current ocular hypotensive medication use or previous glaucoma procedure (laser or surgery).

## Results

### Participants

**Figure 1.**
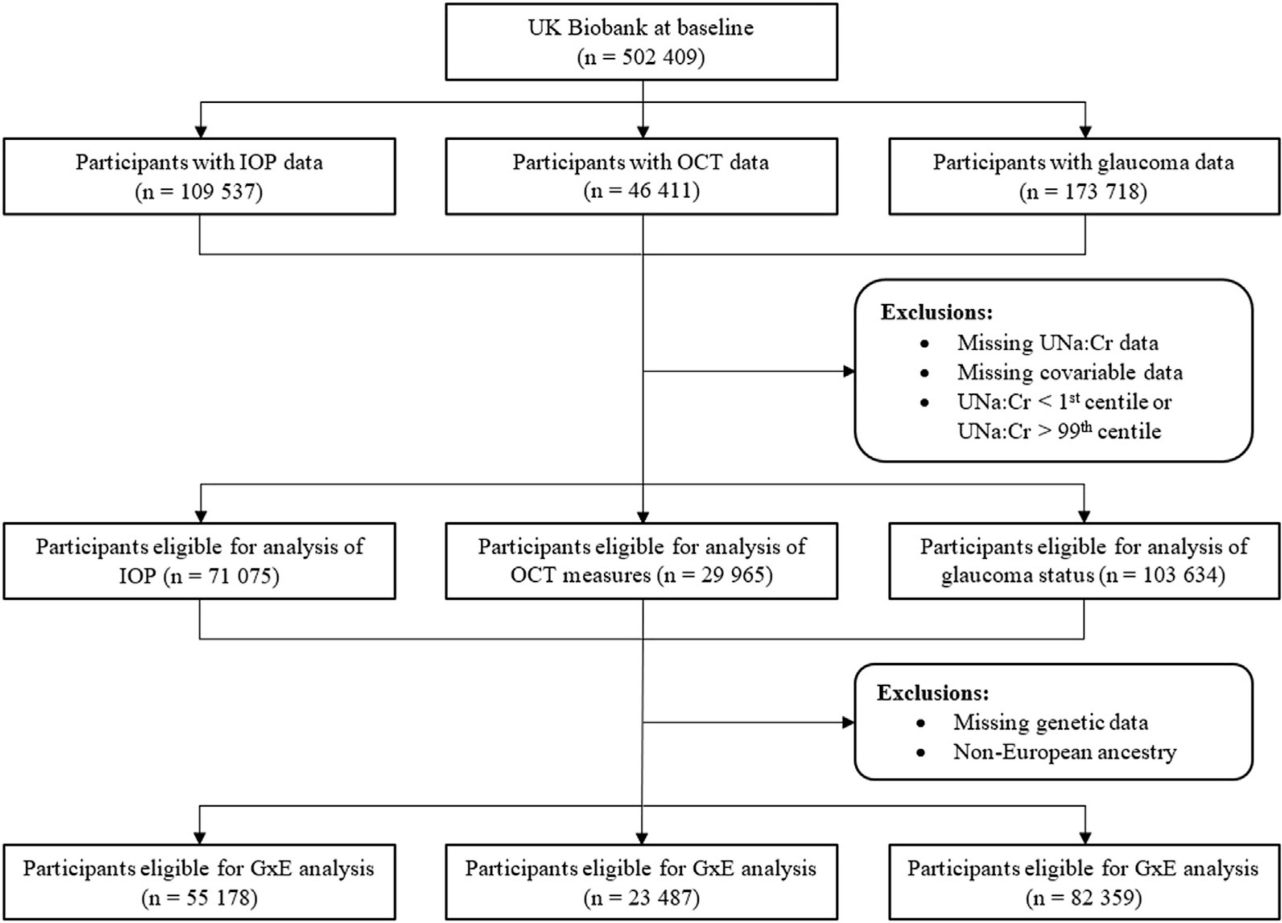
Flow diagram outlining the participant selection process for this study in the UK Biobank. GxE = gene-environment interaction; IOP = intraocular pressure; UNa:Cr = urine sodium:creatinine ratio.

The study flow and participant selection process are summarized in Figure 1. After exclusions for missing data and outliers, 71 075, 29 965, and 103 634 individuals were eligible for the analyses of IOP, OCT derived inner retinal thickness measures, and glaucoma status, respectively. Because there was considerable overlap between cohorts, demographic features and baseline characteristics were largely similar. In keeping with the overall UK Biobank, the mean participant age was 56 to 57 years, with a slight predominance of women (51%–52%), and a majority of White participants (91%–92%) (Table 1). Further restriction to European participants with genetic data left 55 178, 23 487, and 82 359 individuals for the respective gene-environment interaction analyses.

### Urinary Sodium Excretion

**Figure 2.**
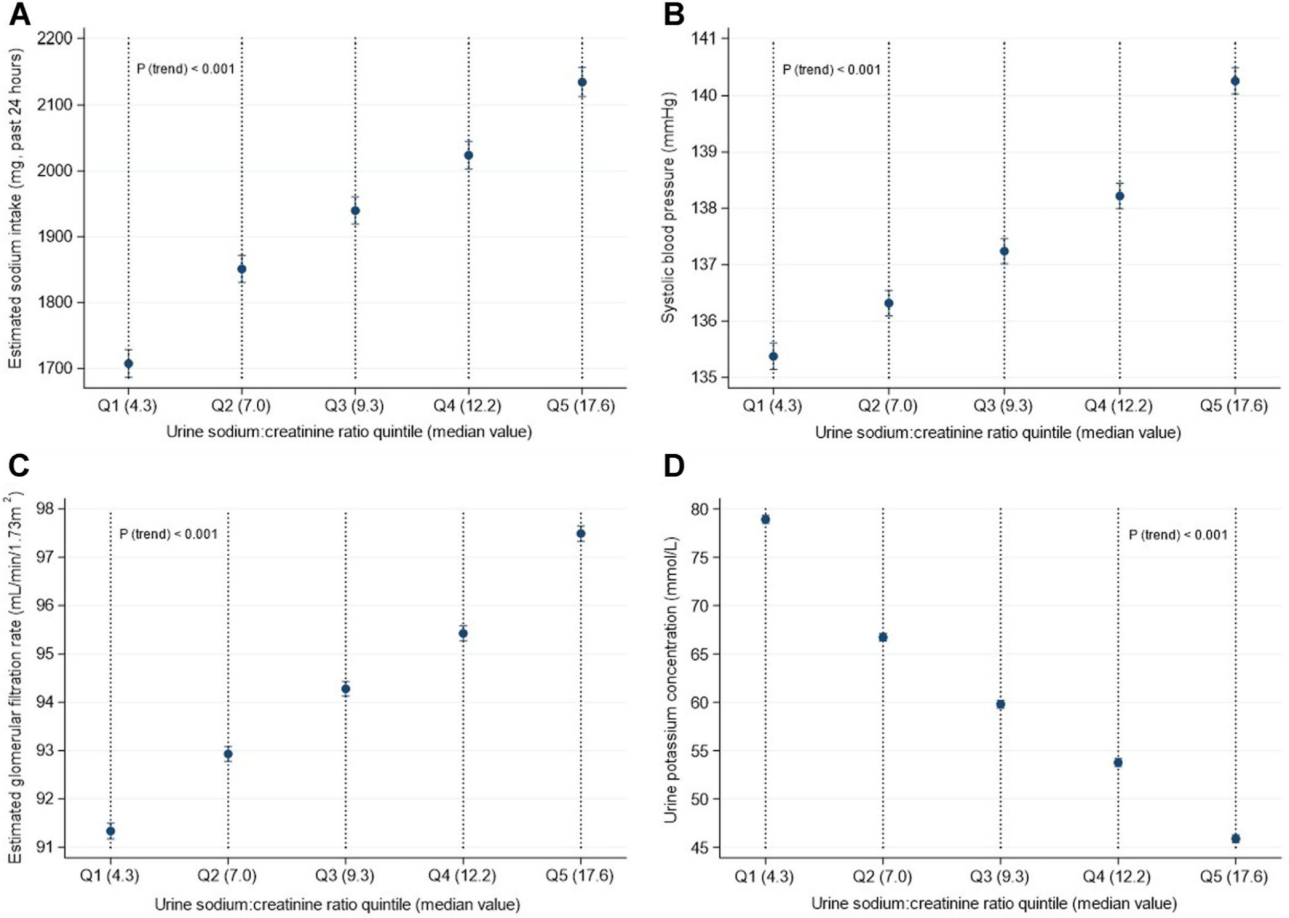
Associations of urinary sodium excretion with (**A**) estimated sodium intake in the past 24 hours, (**B**) systolic blood pressure, (**C**) estimated glomerular filtration rate, and (**D**) urine potassium concentration in UK Biobank participants. Models adjusted for: age (years), sex (women, men), Townsend deprivation index, height (cm), weight (kg), glycated hemoglobin (mmol/mol), total cholesterol (mmol/L), smoking status (never, current, former), alcohol intake (g/day), physical activity (MET-minutes/week), assessment season (Summer, Autumn, Winter, Spring), time of urine collection (morning, afternoon, evening), and urinary potassium concentration (**A–C** only). MET = metabolic equivalent of task; Q = quintile.

Participants characteristics stratified by urine UNa:Cr quintile for individuals included in the analysis of glaucoma status (the largest of the 3 cohorts) are reported in Table 2. There were notable linear trends of estimated 24-hour dietary sodium intake (quintile 1[Q1]: 1773 mg; quintile 5 [Q5]: 2046 mg), SBP (Q1: 135.5 mmHg; Q5: 139.9 mmHg), eGFR (Q1: 91.0 mL/min/1.73 m^2^; Q5: 97.7 mL/ min/1.73 m^2^), and urine potassium concentration (Q1: 79.8 mmol/L; Q5: 44.8 mmol/L) across UNa:Cr quintiles (*P* trend ≤0.001 for all), which persisted after adjustment for all covariables considered in the main analyses (Fig 2). Similar results for the cohorts of IOP and OCT derived inner retinal thickness measures are presented in Tables S3 and S4 (available at www.ophthalmologyglaucoma.org).

### Association with Glaucoma and Related Traits

In maximally adjusted multivariable regression models, a 1 SD increase in UNa:Cr was associated with higher IOP (0.14 mmHg; 95% confidence interval [CI], 0.12–0.17; *P* < 0.001) and greater prevalence of glaucoma (odds ratio, 1.11; 95% CI, 1.07–1.14; *P* < 0.001) but not mRNFL or GCIPL thickness (Table 5, Model A). There was evidence of a dose-response relationship across UNa:Cr quintiles for IOP and glaucoma (*P* trend <0.001 for both) but not for the OCT derived inner retinal parameters (Table 5, Model A). Compared with those in the lowest quintile, those in the highest UNa:Cr quintile had higher IOP (0.45 mmHg; 95% CI, 0.36–0.53; *P* < 0.001) and higher prevalence of glaucoma (odds ratio, 1.30; 95% CI, 1.17–1.45; *P* < 0.001). Further adjustment of the final regression models for SBP resulted in attenuation of the UNa:Cr-IOP association but did not materially affect the relationship with other glaucoma-related outcomes (Table 5, Model B).

### Gene-Environment Interaction Analyses

**Figure 3.**
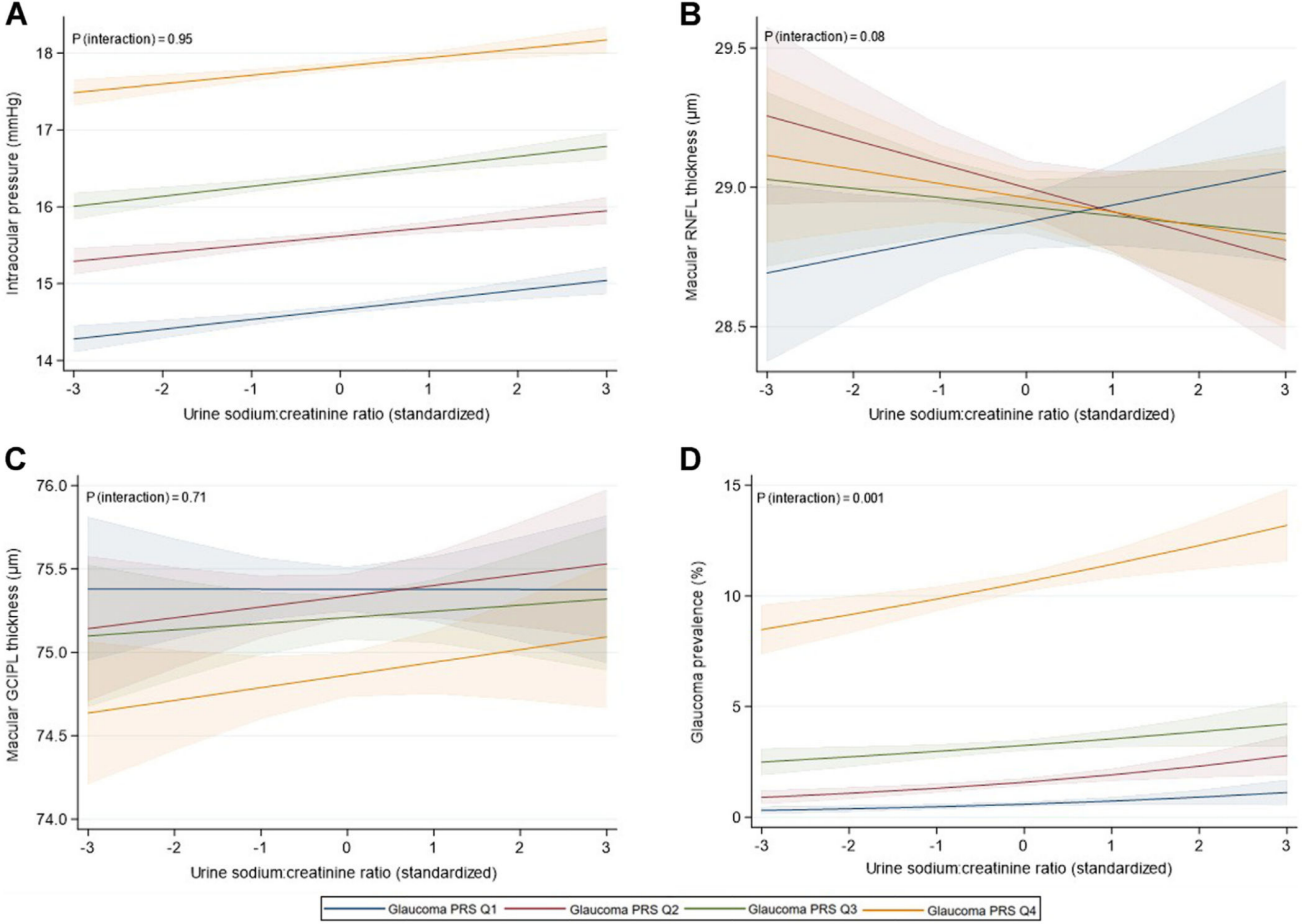
Gene-environment interaction analyses illustrating the effect of the glaucoma PRS on the association of urinary sodium excretion with (**A**) intraocular pressure, (**B**) macular retinal nerve fiber layer thickness, (**C**) macular ganglion cell-inner plexiform layer thickness, and (**D**) glaucoma status in European UK Biobank participants. Models adjusted for: age (years), sex (women, men), Townsend deprivation index, height (cm), weight (kg), glycated hemoglobin (mmol/mol), total cholesterol (mmol/L), smoking status (never, current, former), alcohol intake (g/day), physical activity (MET-minutes/week), assessment season (Summer, Autumn, Winter, Spring), time of urine collection (morning, afternoon, evening), and urinary potassium concentration (mmol/L). GCIPL = ganglion cell-inner plexiform layer; PRS = polygenic risk score; Q = quartile; RNFL = retinal nerve fiber layer.

There was no evidence of a gene-environment interaction for IOP (*P* interaction = 0.95), mRNFL thickness (*P* interaction = 0.32), or GCIPL thickness (*P* interaction = 0.49) (Fig 3A-C). The glaucoma PRS modified the relationship of urinary sodium excretion with glaucoma prevalence (*P* interaction = 0.001); however, with the strongest associations noted in participants at the highest underlying genetic risk (Fig 3D). Although the association between urinary sodium excretion and IOP was the same at all levels of genetic risk, this relationship was not observed for glaucoma. For those in the lowest PRS quartile, urinary sodium excretion was not significantly associated with glaucoma prevalence, with progressively stronger associations noted in subsequent quartiles. For those in the highest PRS quartile, glaucoma prevalence increased from 8.5% to 13.2% across the range of urinary sodium excretion. Further adjustment for SBP did not materially change the results of these analyses (Fig S4, available online at www.ophthalmologyglaucoma.org).

### Sensitivity Analyses

Results for all outcomes were consistent by sex and antihypertensive medication status (Table 6). Associations also persisted when restricting analyses to participants without renal impairment (eGFR >90 ml/min/1.73 m^2^) (Table 6) and results were unchanged when adjusting final models for eGFR or urine microalbumin concentration. Additional adjustment for systemic beta-blocker use and caffeine intake did not materially change the overall results (Table S7, available online at www.ophthalmologyglaucoma.org). Results were also consistent across all alternative glaucoma case definitions, despite substantially fewer cases for ICD-derived definitions (Table S8, available online at www.ophthalmologyglaucoma.org).

## Discussion

In this large population-based study, we investigated the association of urinary sodium excretion, a biomarker of dietary sodium intake, with prevalent glaucoma and various glaucoma-related traits. Overall, consistent adverse dose-response relationships were observed for IOP and glaucoma but not with mRNFL or GCIPL thickness. The relationship with IOP appeared to be partially mediated through systemic blood pressure, while the association with glaucoma prevalence was modified by a glaucoma PRS, with the strongest associations noted in those at the highest underlying genetic risk. Results remained robust to stratified analyses by sex and antihypertensive medication status, and associations also persisted when excluding participants in whom urinary sodium excretion may have been altered from physiological levels by kidney disease.

Urine-based estimations offer an objective and reliable alternative to dietary methods for quantifying sodium intake and the large-scale availability of this biomarker data is a particular strength of the current study.^6,10,12^ Although quantification methods based on multiple 24-hour urine collections are considered the gold standard, numerous technical and practical challenges have limited their uptake in large epidemiological studies. Spot urinary sodium measurements are far easier to obtain, have demonstrated expected associations with blood pressure, and provide a good indication of mean dietary sodium intake on a population level.^12,34^ They are also widely used to estimate 24-hour sodium excretion through a variety of regression-based equations, and, importantly, our analyses included adjustment for all the variables central to these formulae: age, sex, weight, height, urinary creatinine concentration, and urinary potassium concentration.^35–37^ Given that body mass index (BMI) is derived from weight and height, we did not consider it appropriate to additionally adjust for this factor in our regression models. Post hoc adjustment for BMI and adjustment for BMI instead of weight and height did not materially change any of the observed associations. We were also able to validate our exposure measure by assessing associations with relevant dietary data and clinical parameters.

Although our analyses were further strengthened by the large sample size, extensive phenotyping, detailed ocular data, and availability of genetic information in the UK Biobank, it is important to consider certain limitations. Spot urine sodium concentration may reflect recent dietary sodium intake but may not be an accurate representation of long-term salt consumption or capture past changes in dietary behavior. Similarly, the use of these measures is likely to be less accurate than quantification methods based on 24-hour urine collection. We were also limited by our method of glaucoma case ascertainment, which relied on a combination of self-report and ICD codes, although this limitation was partly overcome by our ability to simultaneously assess associations with continuous objective glaucoma-related parameters. Sensitivity analyses also demonstrated consistent results across a variety of alternative glaucoma case definitions. The cross-sectional study design limits our ability to assess temporal relationships and make causal inferences. Although we were able to adjust for multiple important confounders in our analyses, the observed associations may represent residual confounding by unknown or unconsidered factors. Finally, our findings in UK Biobank participants, where > 90% are of self-reported White ethnicity, may not be generalizable to other populations. Multiple studies have demonstrated notable ethnic differences in average dietary intake and urinary excretion of sodium, salt sensitivity, and glaucoma prevalence. It would therefore be important for the findings of this study to be replicated in different cohorts with a greater representation of non-White ethnicities.^3,38,39^

The characteristics of the subset of UK Biobank participants undergoing IOP measurement and OCT imaging have been described in detail previously.^16^ Although largely similar to the overall UK Biobank cohort, those undergoing ophthalmic assessment were more likely to be of non-White ethnicity and have a more positive Townsend deprivation index (indicating greater relative deprivation).^16^ It is also important to note that UK Biobank participants (response rate, 5.5%) were more likely to be older, were more likely to be female, were more likely to live in less socioeconomically deprived areas, and have lower rates of disease when compared to the general UK population (a healthy volunteer effect).^40^ Therefore, although the UK Biobank is not suitable for deriving generalizable estimates of disease prevalence and incidence, the large sample size and heterogeneity of exposures provide for valid assessments of exposure-disease associations that may be generalizable to other populations.^40^

To the best of our knowledge, this is the first population-based study to assess the relationship between urinary sodium excretion and glaucoma. A higher frequency of self-reported dietary salt intake has recently been reported to be adversely associated with prevalent POAG in the TES but only in those using antihypertensive medication.^9^ Important limitations of TES include a relatively small sample size and the use of self-report to assess dietary salt intake, which may have resulted in misclassification bias and limited the investigators’ ability to explore dose-response relationships. Notably, because > 70% of TES participants reported using blood pressure medication, the study may have been underpowered to detect an effect in non-users (292 participants). Alternatively, differences in the exposure (self-reported dietary salt vs. urinary sodium excretion) and population under investigation may mean that the 2 studies are not directly comparable and could account for the disparate results.

These results suggest that urinary sodium excretion and, by extension, dietary sodium intake, may represent a modifiable risk factor for glaucoma, potentially through an IOP-dependent mechanism, and that this effect may be more pronounced in those with a higher glaucoma PRS. Sodium plays a central role in volume homeostasis and increased salt consumption may provoke water retention, leading to a state of high flow in arterial blood vessels.^7^ Fluid overload, increased plasma osmolality, and higher blood pressure, leading to increased aqueous humor production and higher episcleral venous pressures, are plausible biological mechanisms underpinning the relationship between urinary sodium excretion and IOP in this study.^8^ Blood pressure is consistently associated with IOP in epidemiological studies, with a pooled mean IOP of 0.26 mmHg higher per 10 mmHg higher SBP, whereas the acute effect of changes in intravascular fluid volume and concentration have been studied in patients undergoing hemodialysis.^8,41^ It is also possible that vascular and autonomic changes could further influence glaucoma risk through IOP-independent mechanisms.

Current World Health Organization guidelines recommend consuming < 5 g of salt (equivalent to < 2000 mg dietary sodium) daily.^42^ Although we were unable to directly translate UNa:Cr into a measure of dietary intake, only participants in quintile 5 had a mean 24-hour sodium intake exceeding this threshold. While dietary patterns of UK Biobank participants are healthier than those of the general population, the fact that adverse associations were apparent across the range of UNa:Cr values, suggests a continuous relationship rather than one occurring beyond a particular threshold.^43^ This healthy cohort effect is also evidenced by relatively few participants having an eGFR < 60 mL/min/1.73 m^2^. Although renal dysfunction is known to influence urinary sodium excretion, which may therefore not be an accurate reflection of dietary intake in these participants, analyses of this subgroup were likely underpowered.

Despite adverse associations with IOP and glaucoma, urinary sodium excretion was not found to be associated with mRNFL or GCIPL thickness. It is possible that glaucoma-related inner retinal thinning may be masked by sodium-mediated changes in total body water or extracellular fluid volume. For example, higher levels of markers related to body fluid status are correlated with a thicker retinal central subfield in patients with diabetic retinopathy, whereas mean retinal thickness has been shown to decrease significantly after dialysis in patients with end-stage kidney disease.^44,45^

Although adverse associations with IOP were apparent at all levels of genetic risk, progressively stronger associations with prevalent glaucoma were noted in participants with a higher glaucoma PRS. This may suggest that the glaucoma PRS could partly reflect an individual’s susceptibility to IOP-mediated glaucomatous neurodegeneration. Similar interactions have been noted for other dietary factors, including caffeine and alcohol, potentially implicating a combination of environmental exposure and genetically determined functional reserve in the aqueous outflow pathways.^28,32^

It would be important for the results of this study to be replicated in independent cohorts and for the sodium-IOP relationship to be probed further in experimental studies because the presence of an underlying causal association may have important clinical and public health implications and may lead to targeted lifestyle recommendations for glaucoma.^4^ The presence of a significant gene-environment interaction highlights the role that an individual’s underlying genetic architecture may play in determining their susceptibility to lifestyle and environmental risk factors, and raises the possibility of precision nutrition and dietary recommendations based on genomic data in the future.^46^

## Supplementary Material

Figure S4

Table S3

Table S8

Table S4

Table S7

list of consortium members

Supplemental material available at www.ophthalmologyglaucoma.org.

## Figures and Tables

**Table 1.** Baseline Characteristics of Eligible UK Biobank Participants

	Analysis Cohort		
Characteristic (Unit of Measurement)	*IOP*	*OCT*	*Glaucoma*

Sample size, *n*	71 075	29 965	103 634
Age (years)	56.7 (8.1)	56.2 (8.2)	56.9 (8.1)
Sex, *n* (%)			
Women	36 713 (51.7)	15 171 (50.6)	52 991 (51.1)
Men	34 362 (48.3)	14 794 (49.4)	50 643 (48.9)
Ethnicity, *n* (%)			
White	64 762 (91.1)	27 655 (92.3)	95 682 (92.3)
Asian	2760 (3.9)	907 (3.0)	3457 (3.3)
Black	1970 (2.8)	737 (2.5)	2401 (2.3)
Other/mixed	1583 (2.2)	666 (2.2)	2094 (2.0)
Townsend deprivation index	−1.1 (2.9)	−1.1 (2.9)	−1.1 (3.0)
Height (cm)	169.2 (9.3)	169.5 (9.2)	169.1 (9.3)
Weight (kg)	78.2 (15.9)	78.4 (15.7)	78.3 (15.9)
Body mass index (kg/m^2^)	27.2 (4.6)	27.2 (4.6)	27.3 (4.7)
Systolic blood pressure (mmHg)	137.0 (18.2)	136.7 (18.3)	137.5 (18.4)
HbA1c (mmol/mol)	36.1 (6.6)	35.9 (6.6)	36.2 (7.0)
Total cholesterol (mmol/L)	5.7 (1.1)	5.7 (1.1)	5.7 (1.1)
Smoking status, *n* (%)			
Never smoker	39 265 (55.2)	16 316 (54.5)	56 107 (54.1)
Current smoker	6857 (9.7)	2916 (9.7)	10 311 (10.0)
Former smoker	24 953 (35.1)	10 733 (35.8)	37 216 (35.9)
Alcohol intake (g/week)	107.5 (129.4)	109.0 (128.5)	114.1 (133.3)
Physical activity (MET-hours/week)	44.7 (44.6)	45.2 (45.1)	44.5 (44.8)
Urine sodium concentration (mmol/L)	72.8 (40.7)	72.3 (40.4)	73.9 (41.7)
Urine potassium concentration (mmol/L)	59.8 (31.4)	60.0 (31.5)	61.0 (32.2)
Urine creatinine concentration (mmol/L)	8.4 (5.2)	8.5 (5.3)	8.6 (5.4)
Urine sodium:creatinine ratio (mmol:mmol)	10.2 (5.3)	10.0 (5.2)	10.3 (5.3)
Quintile 1, range	<5.7	<5.6	<5.7
Quintile 2, range	5.7-8.1	5.6-7.9	5.7-8.1
Quintile 3, range	8.1-10.6	7.9-10.4	8.1-10.7
Quintile 4, range	10.6-14.2	10.4-13.9	10.7-14.3
Quintile 5, range	>14.2	>13.9	>14.3
eGFR (mL/min/1.73 m^2^)	94.4 (12.8)	94.4 (12.7)	94.3 (13.0)
Intraocular pressure (mmHg)	16.1 (3.4)	–	–
mRNFL thickness (μm)	–	28.9 (3.8)	–
GCIPL thickness (μm)	–	75.2 (5.2)	–
Glaucoma prevalence, *n* (%)	–	–	4045 (3.9)

eGFR = estimated glomerular filtration rate; GCIPL = ganglion cell-inner plexiform layer; HbA1c = glycated hemoglobin; IOP = intraocular pressure; MET = metabolic equivalent of task; mRNFL = macular retinal nerve fiber layer.

All values represent mean (standard deviation), unless otherwise specified.

**Table 2.** Baseline Characteristics of Eligible UK Biobank Participants by Urine Sodium:Creatinine Ratio Quintile (Glaucoma Cohort)

	Urine Sodium:Creatinine Ratio Quintile (mmol:mmol) (n = 103 634)					
Characteristic (Unit of Measurement)	*Quintile 1 (*<*5.7)*	*Quintile 2 (5.7–8.1)*	*Quintile 3 (8.1*–*10.7)*	*Quintile 4 (10.7*–*14.3)*	*Quintile 5 (*>*14.3)*	*P* (Trend)

Age, years	57.4 (8.0)	57.0 (8.1)	56.8 (8.1)	56.8 (8.1)	56.7 (8.2)	<0.001
Sex, women, *n* (%)	8888 (42.9)	9356 (45.1)	10 099 (48.7)	11 156 (53.8)	13 492 (65.1)	<0.001
Ethnicity, White, *n* (%)	19 344 (93.2)	19 446 (93.8)	19 232 (92.8)	19 126 (92.3)	18 534 (89.4)	<0.001
Townsend deprivation index	−1.2 (3.0)	−1.2 (3.0)	−1.2 (3.0)	−1.1 (3.0)	−0.9 (3.0)	<0.001
Height, cm	170.9 (9.3)	170.4 (9.2)	169.6 (9.2)	168.4 (9.0)	166.2 (8.9)	<0.001
Weight, kg	81.2 (16.1)	79.6 (15.6)	78.5 (15.7)	77.3 (15.6)	74.8 (15.6)	<0.001
Body mass index, kg/m^2^	27.7 (4.7)	27.3 (4.5)	27.2 (4.5)	27.2 (4.6)	27.0 (4.8)	<0.001
Systolic blood pressure, mmHg	135.5 (17.8)	136.4 (18.0)	137.3 (18.0)	138.3 (18.4)	139.9 (19.3)	<0.001
HbA1c, mmol/mol	36.4 (7.5)	36.2 (7.0)	36.2 (6.8)	36.1 (6.6)	36.3 (6.9)	0.32
Total cholesterol, mmol/L	5.6 (1.2)	5.7 (1.1)	5.7 (1.1)	5.7 (1.1)	5.7 (1.1)	<0.001
Smoking status, current smoker, *n* (%)	2150 (10.4)	2072 (10.0)	1993 (9.6)	2026 (9.8)	2070 (10.0)	0.16
Alcohol intake, g/week	123.7 (144.8)	120.9 (137.7)	115.1 (132.3)	109.6 (126.0)	101.3 (123.4)	< 0.001
Physical activity, MET-hours/week	41.2 (42.5)	42.9 (43.0)	45.3 (45.6)	45.9 (45.8)	47.1 (47.0)	< 0.001
Urine sodium concentration, mmol/L	51.5 (26.5)	67.6 (34.4)	76.4 (40.0)	83.1 (44.2)	91.0 (48.1)	< 0.001
Urine potassium concentration, mmol/L	79.8 (36.2)	67.2 (32.2)	59.9 (29.4)	53.4 (27.3)	44.8 (23.0)	< 0.001
Urine creatinine concentration. mmol/L	13.0 (6.5)	9.8 (5.0)	8.2 (4.3)	6.8 (3.6)	5.0 (2.8)	< 0.001
eGFR, mL/min/1.73 m^2^	91.0 (14.1)	92.9 (13.1)	94.4 (12.6)	95.5 (12.3)	97.7 (11.8)	< 0.001
Intraocular pressure, mmHg*	15.9 (3.4)	16.0 (3.4)	16.1 (3.4)	16.1 (3.4)	16.1 (3.4)	< 0.001
mRNFL thickness, μm^†^	28.9 (3.9)	29.0 (3.8)	28.9 (3.8)	29.0 (3.8)	28.9 (3.8)	0.49
GCIPL thickness, μm^‡^	75.1 (5.3)	75.2 (5.2)	75.3 (5.3)	75.3 (5.2)	75.3 (5.1)	0.004
Glaucoma prevalence, *n* (%)	845 (4.1)	766 (3.7)	793 (3.8)	801 (3.9)	840 (4.1)	0.13
Estimated sodium intake, mg, 24-hr recall^§^	1773 (871)	1888 (881)	1945 (932)	1997 (929)	2046 (985)	< 0.001

eGFR = estimated glomerular filtration rate; GCIPL = ganglion cell-inner plexiform layer; HbA1c = glycated hemoglobin; MET = metabolic equivalent of task; mRNFL = macular retinal nerve fiber layer. All values represent mean (standard deviation), unless otherwise specified. Boldface indicates *P* values <0.05.

* n = 70 793.

† n = 29 616.

‡ n = 29 532.

§ n = 35 566.

**Table 5.** Results of Multivariable Regression Analyses for the Association of Urinary Sodium Excretion with Glaucoma and Related Traits

	Intraocular Pressure (mmHg) (n = 71 075)			mRNFL Thickness (μm) (n = 29 660)			GCIPL Thickness (μm) (n = 29 577)			Glaucoma Prevalence (%) (n = 103 634)		
Urine Sodium: Creatinine Ratio	*Beta*	*95% CI*	P *Value*	*Beta*	*95% CI*	P *Value*	*Beta*	*95% CI*	P *Value*	*OR*	*95% CI*	P *Value*

Model A (without SBP)*												
Continuous												
Per SD increase Quintiles^†^	0.14	0.12–0.17	**< 0.001**	−0.03	−0.08, 0.01	0.17	0.03	−0.03, 0.10	0.32	1.11	1.07, 1.14	**< 0.001**
Quintile 1		Reference			Reference			Reference			Reference	
Quintile 2	0.15	0.07–0.23	**< 0.001**	0.06	−0.08 to 0.20	0.39	0.09	−0.10 to 0.27	0.37	0.99	0.90, 1.10	0.91
Quintile 3	0.30	0.22–0.38	**< 0.001**	−0.03	−0.17 to 0.11	0.68	0.10	−0.09 to 0.29	0.29	1.10	0.99, 1.21	0.09
Quintile 4	0.33	0.25–0.42	**< 0.001**	−0.03	−0.17 to 0.11	0.66	0.11	−0.08 to 0.30	0.26	1.15	1.03, 1.28	**0.009**
Quintile 5	0.45	0.36–0.53	**< 0.001**	−0.08	−0.22 to 0.07	0.30	0.16	−0.04 to 0.36	0.12	1.30	1.17, 1.45	**< 0.001**
*P* (trend)			**< 0.001**			0.14			0.15			**< 0.001**
Model B (with SBP)^‡^												
Continuous												
Per SD increase Quintiles^†^	0.09	0.06–0.12	**< 0.001**	−0.03	−0.08 to 0.02	0.20	0.05	−0.02 to 0.11	0.17	1.10	1.06, 1.14	**< 0.001**
Quintile 1		Reference			Reference			Reference			Reference	
Quintile 2	0.12	0.04–0.20	**0.002**	0.06	−0.08 to 0.20	0.38	0.09	−0.09 to 0.28	0.33	0.99	0.90 to 1.10	0.87
Quintile 3	0.24	0.16–0.32	**< 0.001**	−0.03	−0.17 to 0.11	0.69	0.11	−0.08 to 0.30	0.24	1.09	0.98 to 1.21	0.10
Quintile 4	0.24	0.16–0.32	**< 0.001**	−0.03	−0.17 to 0.11	0.68	0.13	−0.06 to 0.32	0.18	1.14	1.03 to 1.27	**0.013**
Quintile 5	0.30	0.21–0.38	**< 0.001**	−0.07	−0.22 to 0.07	0.33	0.19	−0.01 to 0.39	0.06	1.29	1.16 to 1.44	**< 0.001**
*P* (trend)			**< 0.001**			0.16			0.07			**<0.001**

CI =confidence interval; GCIPL = ganglion cell-inner plexiform layer; mRNFL = macular retinal nerve fiber layer; OR = odds ratio; SBP = systolic blood pressure; SD, standard deviation. Boldface indicates *P* values <0.05.

* Model A adjusted for: age (years), sex (women, men), ethnicity (White, Asian, Black, other/mixed), Townsend deprivation index, height (cm), weight (kg), glycated hemoglobin (mmol/mol), total cholesterol (mmol/L), smoking status (never, current, former), alcohol intake (g/day), physical activity (metabolic equivalent of task-minutes/week), assessment season (Summer, Autumn, Winter, Spring), time of urine collection (morning, afternoon, evening), and urinary potassium concentration (mmol/L).

† Details of urine sodium:creatinine ratio quintiles for each cohort are available in Table 1.

‡ Model B adjusted for: as for model A, plus systolic blood pressure (mmHg).

**Table 6.** Results of Multivariable Regression Analyses for the Association of Urinary Sodium Excretion with Glaucoma and Related Traits, Stratified by Renal Function, Antihypertensive Medication Use, and Sex

Urine Sodium:Creatinine Ratio (Per SD Increase)	Intraocular Pressure (mmHg)				mRNFL Thickness (μm)				GCIPL Thickness (μm)				Glaucoma Prevalence (%)			
	n	*Beta*	95% *CI*	P *Value*	n	* Beta*	*95% CI*	P *Value*	n	*Beta*	* 95% CI*	P *Value*	n	*OR*	*95% CI*	P *Value*

Model A (without SBP)*																
eGFR (mL/min/1.73 m^2^)																
≥ 90	48 633	0.16	0.13–0.19	***<* 0.001**	20 295	−0.05	−0.10 to 0.01	0.10	20 243	0.04	−0.04 to 0.12	0.29	70970	1.13	1.09– 1.18	***<* 0.001**
60 to < 90	21411	0.09	0.04–0.14	**0.001**	8977	0.01	−0.08 to 0.09	0.90	8948	−0.02	−0.15 to 0.09	0.63	31 043	1.04	0.98– 1.10	0.20
< 60	965	0.13	−0.12 to 0.38	0.32	355	−0.17	−0.64 to 0.30	0.48	354	−0.15	−0.79 to 0.48	0.63	1534	1.00	0.78– 1.28	0.99
Antihypertensive use																
No	56 702	0.14	0.11–0.17	***<* 0.001**	23 977	−0.04	−0.09 to 0.02	0.18	23 916	0.04	−0.03 to 0.11	0.25	81 609	1.11	1.06– 1.16	***<* 0.001**
Yes	14373	0.15	0.09–0.22	***<* 0.001**	5683	0.01	−0.10 to 0.12	0.88	5661	0.05	−0.10 to 0.20	0.54	22 025	1.10	1.04– 1.18	**0.003**
Sex																
Women	36 713	0.12	0.08–0.15	***<* 0.001**	15012	−0.02	−0.08 to 0.05	0.65	15 009	0.00	−0.09 to 0.09	0.99	52 991	1.10	1.05– 1.16	***<* 0.001**
Men	34 362	0.18	0.14–0.22	***<* 0.001**	14648	−0.05	−0.12 to 0.01	0.10	14 568	0.07	−0.02 to 0.17	0.12	50 643	1.10	1.06– 1.15	***<* 0.001**
Model B (with SBP)^†^																
eGFR (mL/min/1.73 m^2^)																
≥ 90	48 633	0.10	0.07–0.13	***<* 0.001**	20 295	−0.05	−0.10 to 0.01	0.12	20 243	0.05	−0.02 to 0.13	0.18	70970	1.13	1.08– 1.18	***<* 0.001**
60 to < 90	21 411	0.03	−0.02 to 0.08	0.20	8977	0.00	−0.08 to 0.09	0.93	8948	−0.01	−0.13 to 0.10	0.81	31 043	1.04	0.98– 1.10	0.25
< 60	965	0.14	−0.11 to 0.39	0.27	355	−0.20	−0.68 to 0.28	0.41	354	−0.09	−0.73 to 0.56	0.79	1534	1.00	0.78– 1.28	0.99
Antihypertensive use																
No	56 702	0.09	0.06–0.11	***<* 0.001**	23 977	−0.04	−0.09 to 0.02	0.19	23 916	0.05	−0.02 to0.13	0.13	81 609	1.10	1.06– 1.15	***<* 0.001**
Yes	14 373	0.11	0.05–0.18	**0.001**	5683	0.01	−0.10 to 0.12	0.84	5661	0.05	−0.10 to 0.20	0.47	22 025	1.11	1.04– 1.18	**0.002**
Sex																
Women	36 713	0.06	0.02–0.09	**0.001**	15012	−0.02	−0.08 to 0.05	0.61	15 009	0.01	−0.07 to 0.10	0.76	52 991	1.09	1.04– 1.15	**0.001**
Men	34 362	0.13	0.09–0.17	***<* 0.001**	14 648	−0.05	−0.12 to 0.02	0.14	14 568	0.09	−0.01 to 0.18	0.06	50 643	1.11	1.06– 1.15	***<* 0.001**

CI = confidence interval; eGFR = estimated glomerular filtration rate; GCIPL = ganglion cell-inner plexiform layer; mRNFL = macular retinal nerve fiber layer; OR = odds ratio; SBP = systolic blood pressure; SD = standard deviation. Boldface indicates *P* values <0.05.

* Model A adjusted for: age (years), sex (women, men), ethnicity (White, Asian, Black, other), Townsend deprivation index, height (cm), weight (kg), glycated hemoglobin (mmol/mol), total cholesterol (mmol/L), smoking status (never, current, former), alcohol intake (g/day), physical activity (metabolic equivalent of task-minutes/week), assessment season (Summer, Autumn, Winter, Spring), time of urine collection (morning, afternoon, evening), and urinary potassium concentration (mmol/L).

† Model B adjusted for: as for model A, plus systolic blood pressure (mmHg).

## Abbreviations and Acronyms:

BMI: body mass index
CI: confidence interval
CV: coefficient of variation
eGFR: estimated glomerular filtration rate
GCIPL: ganglion cell-inner plexiform layer
ICD: International Classification of Diseases
IOP: intraocular pressure
mRNFL: macular retinal nerve fiber layer
POAG: primary open-angle glaucoma
PRS: polygenic risk score
SBP: systolic blood pressure
SD: standard deviation
TES: Thessaloniki Eye Study
UNa:Cr: urine sodium:creatinine ratio
