# Supplementary material for: The Association of Urinary Sodium Excretion with Glaucoma and Related Traits in a Large United Kingdom Population
Source: Ophthalmol Glaucoma. Author manuscript; Available in PMC 2025 Jun 18. (PMC12174990; doi:10.1016/j.ogla.2024.04.010)
Supplement: Figure S4 [file NIHMS2083578-supplement-Figure_S4.pdf]

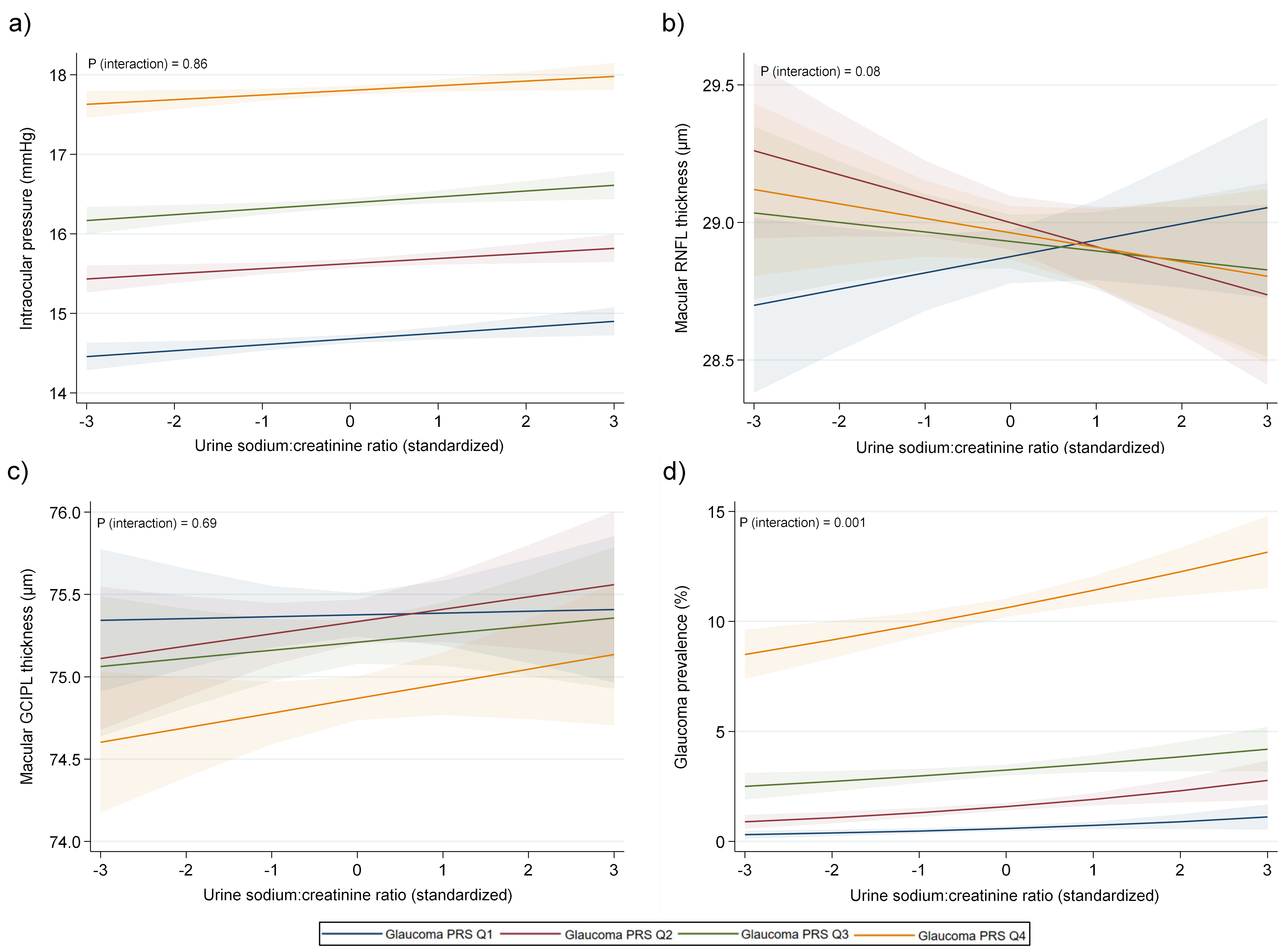
a)
P (interaction) = 0.86
Intraocular pressure (mmHg)
18
17
16
15
14
-3
-2
-1
0
1
2
3
Urine sodium:creatinine ratio (standardized)
b)
P (interaction) = 0.08
Macular RNFL thickness (μm)
29.5
29.0
28.5
Urine sodium:creatinine ratio (standardized)
c)
P (interaction) = 0.69
Macular GCIPL thickness (μm)
76.0
75.5
75.0
74.5
74.0
Urine sodium:creatinine ratio (standardized)
d)
P (interaction) = 0.001
Glaucoma prevalence (%)
15
10
5
0
Urine sodium:creatinine ratio (standardized)
Glaucoma PRS Q1
Glaucoma PRS Q2
Glaucoma PRS Q3
Glaucoma PRS Q4

Figure S4. Gene-environment interaction analyses illustrating the effect of the glaucoma PRS on the association of urinary sodium excretion with **a)** intraocular pressure, **b)** macular retinal nerve fibre layer thickness, **c)** macular ganglion cell-inner plexiform layer thickness, and **d)** glaucoma status in European UK Biobank participants, with additional adjustment for systolic blood pressure

Models adjusted for: age (years), sex (women, men), Townsend deprivation index, height (cm), weight (kg), glycated hemoglobin (mmol/mol), total cholesterol (mmol/L), smoking status (never, current, former), alcohol intake (g/day), physical activity (MET-minutes/week), assessment season (Summer, Autumn, Winter, Spring), time of urine collection (morning, afternoon, evening), urinary potassium concentration (mmol/L), and systolic blood pressure (mmHg). RNFL, retinal nerve fiber layer; GCIPL, ganglion cell-inner plexiform layer; PRS, polygenic risk score; Q, quartile.
