# Supplementary material for: The Association of Urinary Sodium Excretion with Glaucoma and Related Traits in a Large United Kingdom Population
Source: Ophthalmol Glaucoma. Author manuscript; Available in PMC 2025 Jun 18. (PMC12174990; doi:10.1016/j.ogla.2024.04.010)
Supplement: Table S3 [file NIHMS2083578-supplement-Table_S3.pdf]

Table S3. Baseline characteristics of eligible UK Biobank participants by urine sodium:creatinine ratio quintile (intraocular pressure cohort)

**Characteristic (unit of measurement)**	**Urine sodium:creatinine ratio quintile (mmol:mmol) (*n* = 71 075)**					***P* (trend)**
	**Quintile 1 (<576)**	**Quintile 2 (5.7–8.1)**	**Quintile 3 (8.1–10.6)**	**Quintile 4 (10.6–14.2)**	**Quintile 5 (>14.2)**	
Age (years)	57.1 (8.1)	56.8 (8.1)	56.5 (8.1)	56.6 (8.1)	56.3 (8.2)	**<0.001**
Sex (women), *n* (%)	6 091 (42.9)	6 448 (45.4)	6 972 (49.1)	7 757 (54.6)	9 445 (66.4)	**<0.001**
Ethnicity (White), *n* (%)	13 142 (92.5)	13 209 (92.9)	13 018 (91.6)	12 936 (91.0)	12 457 (87.6)	**<0.001**
Townsend deprivation index	-1.1 (3.0)	-1.2 (2.9)	-1.1 (2.9)	-1.0 (2.9)	-0.9 (2.9)	**<0.001**
Height (cm)	171.1 (9.2)	170.4 (9.2)	169.7 (9.2)	168.5 (9.0)	166.1 (8.8)	**<0.001**
Weight (kg)	81.3 (16.1)	79.6 (15.6)	78.4 (15.5)	77.3 (15.7)	74.5 (15.4)	**<0.001**
Body mass index (kg/m^2)	27.7 (4.7)	27.3 (4.5)	27.1 (4.5)	27.1 (4.7)	26.9 (4.8)	**<0.001**
Systolic blood pressure (mmHg)	135.0 (17.7)	136.0 (17.8)	136.8 (17.9)	137.9 (18.4)	139.2 (19.2)	**<0.001**
HbA1c (mmol/mol)	36.2 (7.3)	36.1 (6.6)	36.0 (6.6)	36.0 (6.3)	36.1 (6.4)	0.22
Total cholesterol (mmol/L)	5.6 (1.1)	5.7 (1.1)	5.7 (1.1)	5.7 (1.1)	5.7 (1.1)	**<0.001**
Smoking status (current smoker), *n* (%)	1 441 (10.1)	1 384 (9.7)	1 324 (9.3)	1 360 (9.6)	1 348 (9.5)	0.42
Alcohol intake (g/week)	117.0 (139.7)	115.1 (135.1)	108.4 (128.1)	103.2 (122.1)	93.9 (119.4)	**<0.001**
Physical activity (MET-hours/week)	41.5 (42.3)	43.1 (42.7)	45.5 (45.3)	46.0 (45.7)	47.4 (46.4)	**<0.001**
Urine sodium concentration (mmol/L)	50.8 (25.8)	66.7 (33.7)	75.3 (39.3)	81.1 (43.1)	89.5 (47.0)	**<0.001**
Urine potassium concentration (mmol/L)	78.2 (35.4)	66.2 (31.5)	58.7 (28.6)	52.3 (26.3)	43.8 (22.2)	**<0.001**
Urine creatinine concentration (mmol/L)	12.8 (6.3)	9.7 (4.9)	8.1 (4.2)	6.7 (3.6)	5.0 (2.7)	**<0.001**
eGFR (mL/min/1.73m^2)	91.2 (13.8)	92.8 (13.0)	94.5 (12.4)	95.5 (12.2)	97.8 (11.6)	**<0.001**
Intraocular pressure (mmHg)	15.9 (3.4)	16.0 (3.4)	16.1 (3.5)	16.1 (3.4)	16.2 (3.4)	**<0.001**
mRNFL thickness (µm) [a]	28.9 (3.9)	29.0 (3.8)	28.9 (3.8)	29.0 (3.8)	28.9 (3.8)	0.67
GCIPL thickness (µm) [b]	75.1 (5.3)	75.2 (5.2)	75.3 (5.3)	75.3 (5.2)	75.4 (5.1)	**0.003**
Glaucoma prevalence, *n* (%) [c]	238 (1.7)	178 (1.3)	188 (1.3)	212 (1.5)	183 (1.3)	**0.020**
Estimated sodium intake (mg, 24-hour recall) [d]	1 779 (866)	1 894 (887)	1 941 (914)	2 001 (929)	2 045 (987)	**<0.001**

All values represent mean (standard deviation), unless otherwise specified. [a] *n* = 28 807. [b] *n* = 28 725. [c] *n* = 70 793. [d] *n* = 31 449.
HbA1c, glycated hemoglobin; MET, metabolic equivalent of task; eGFR, estimated glomerular filtration rate; mRNFL, macular retinal nerve fiber layer; GCIPL, ganglion cell-inner plexiform layer.
