# Supplementary material for: The Association of Urinary Sodium Excretion with Glaucoma and Related Traits in a Large United Kingdom Population
Source: Ophthalmol Glaucoma. Author manuscript; Available in PMC 2025 Jun 18. (PMC12174990; doi:10.1016/j.ogla.2024.04.010)
Supplement: Table S8 [file NIHMS2083578-supplement-Table_S8.pdf]

Table S8. Results of multivariable regression analyses for the association of urinary sodium excretion with alternative glaucoma case definitions

Urine sodium:creatinine ratio	Self-reported glaucoma (3 843 cases)			ICD-coded glaucoma (617 cases)			ICD-coded POAG (263 cases)			Glaucoma medication/procedure (2 433 cases)		
	**OR**	**95% CI**	***P*-value**	**OR**	**95% CI**	***P*-value**	**OR**	**95% CI**	***P*-value**	**OR**	**95% CI**	***P*-value**
**Model A (without SBP)** [a]												
Continuous												
Per SD increase	1.11	1.07, 1.15	**<0.001**	1.09	1.00, 1.19	**0.042**	1.15	1.01, 1.31	**0.040**	1.11	1.07, 1.16	**<0.001**
Quintiles [b]												
Quintile 1	Reference			Reference			Reference			Reference		
Quintile 2	1.01	0.91, 1.12	0.86	1.16	0.90, 1.50	0.26	1.28	0.87, 1.88	0.20	1.15	1.02, 1.31	**0.029**
Quintile 3	1.09	0.98, 1.21	0.12	1.26	0.97, 1.63	0.08	0.97	0.64, 1.48	0.90	1.18	1.04, 1.35	**0.013**
Quintile 4	1.16	1.04, 1.29	**0.006**	1.23	0.94, 1.61	0.13	1.45	0.98, 2.16	0.07	1.22	1.07, 1.40	**0.004**
Quintile 5	1.33	1.19, 1.48	**<0.001**	1.33	1.01, 1.75	**0.045**	1.55	1.02, 2.36	**0.040**	1.39	1.21, 1.60	**<0.001**
P (trend)			**<0.001**			**0.050**			**0.038**			**<0.001**
**Model B (with SBP)** [c]												
Continuous												
Per SD increase	1.11	1.07, 1.15	**<0.001**	1.09	1.00, 1.19	**0.045**	1.13	0.99, 1.29	0.06	1.11	1.06, 1.16	**<0.001**
Quintiles [b]												
Quintile 1	Reference			Reference			Reference			Reference		
Quintile 2	1.01	0.91, 1.12	0.89	1.16	0.90, 1.50	0.26	1.27	0.87, 1.87	0.21	1.15	1.01, 1.31	**0.032**
Quintile 3	1.08	0.98, 1.20	0.13	1.26	0.97, 1.63	0.08	0.96	0.63, 1.47	0.86	1.18	1.03, 1.34	**0.016**
Quintile 4	1.16	1.04, 1.29	**0.008**	1.23	0.94, 1.61	0.13	1.43	0.96, 2.13	0.08	1.21	1.06, 1.39	**0.006**
Quintile 5	1.31	1.17, 1.47	**<0.001**	1.32	1.00, 1.75	**0.047**	1.50	0.99, 2.29	0.06	1.37	1.19, 1.58	**<0.001**
P (trend)			**<0.001**			0.05			0.06			**<0.001**

[a] Model A adjusted for: age (years), sex (women, men), ethnicity (White, Asian, Black, Other/Mixed), Townsend deprivation index, height (cm), weight (kg), glycated hemoglobin (mmol/mol), total cholesterol (mmol/L), smoking status (never, current, former), alcohol intake (g/day), physical activity (MET-minutes/week), assessment season (Summer, Autumn, Winter, Spring), time of urine collection (morning, afternoon, evening), urinary potassium concentration (mmol/L), systemic beta-blocker use (no, yes), and caffeine intake (mg/day). [b] Details of urine sodium:creatinine ratio quintiles for each cohort are available in Table 1. [c] Model B adjusted for: as for Model A, plus systolic blood pressure (mmHg).

mRNFL, macular retinal nerve fiber layer; GCIPL, ganglion cell-inner plexiform layer; CI, confidence interval; OR, odds ratio; SD, standard deviation; SBP, systolic blood pressure; ICD, International Classification of Diseases (10th edition); POAG, primary open-angle glaucoma.
