# Supplementary material for: The Association of Urinary Sodium Excretion with Glaucoma and Related Traits in a Large United Kingdom Population
Source: Ophthalmol Glaucoma. Author manuscript; Available in PMC 2025 Jun 18. (PMC12174990; doi:10.1016/j.ogla.2024.04.010)
Supplement: Table S4 [file NIHMS2083578-supplement-Table_S4.pdf]

Table S4. Baseline characteristics of eligible UK Biobank participants by urine sodium:creatinine ratio quintile (optical coherence tomography cohort)

**Characteristic (unit of measurement)**	**Urine sodium:creatinine ratio quintile (mmol:mmol) (*n* = 29 965)**					***P* (trend)**
	**Quintile 1 (<5.6)**	**Quintile 2 (5.6–7.9)**	**Quintile 3 (7.9–10.4)**	**Quintile 4 (10.4–13.9)**	**Quintile 5 (>13.9)**	
Age (years)	56.7 (8.1)	56.2 (8.1)	55.9 (8.2)	56.1 (8.1)	56.0 (8.3)	**<0.001**
Sex (women), *n* (%)	2 526 (42.2)	2 660 (44.4)	2 872 (47.9)	3 200 (53.4)	3 913 (65.3)	**<0.001**
Ethnicity (White), *n* (%)	5 590 (93.3)	5 636 (94.0)	5 546 (92.5)	5 519 (92.1)	5 364 (89.5)	**<0.001**
Townsend deprivation index	-1.2 (2.9)	-1.2 (2.9)	-1.2 (2.9)	-1.0 (2.9)	-0.9 (2.9	**<0.001**
Height (cm)	171.2 (9.2)	170.8 (9.1)	170.2 (9.1)	168.8 (9.1)	166.4 (8.8)	**<0.001**
Weight (kg)	81.3 (16.0)	80.0 (15.6)	78.7 (15.5)	77.3 (15.5)	74.7 (15.3)	**<0.001**
Body mass index (kg/m^2)	27.7 (4.6)	27.3 (4.5)	27.1 (4.4)	27.1 (4.6)	26.9 (4.7)	**<0.001**
Systolic blood pressure (mmHg)	134.6 (17.7)	135.5 (17.6)	136.3 (17.8)	137.8 (18.4)	139.2 (19.4)	**<0.001**
HbA1c (mmol/mol)	36.0 (7.5)	35.8 (6.3)	35.9 (6.9)	35.7 (6.1)	35.9 (6.3)	0.20
Total cholesterol (mmol/L)	5.6 (1.1)	5.7 (1.1)	5.7 (1.1)	5.7 (1.1)	5.8 (1.1)	**<0.001**
Smoking status (current smoker), *n* (%)	618 (10.3)	593 (9.9)	552 (9.2)	601 (10.0)	552 (9.2)	0.28
Alcohol intake (g/week)	117.0 (139.0)	114.7 (127.7)	110.0 (127.8)	105.5 (124.8)	97.7 (121.7)	**<0.001**
Physical activity (MET-hours/week)	41.8 (42.9)	43.7 (43.6)	46.8 (46.9)	45.6 (45.0)	47.8 (46.7)	**<0.001**
Urine sodium concentration (mmol/L)	50.2 (25.7)	66.0 (33.1)	75.1 (38.8)	81.6 (42.9)	88.5 (46.8)	**<0.001**
Urine potassium concentration (mmol/L)	77.7 (35.2)	66.4 (31.7)	59.1 (28.9)	52.9 (26.8)	43.7 (22.0)	**<0.001**
Urine creatinine concentration (mmol/L)	12.8 (6.4)	9.8 (4.9)	8.3 (4.3)	6.9 (3.6)	5.0 (2.8)	**<0.001**
eGFR (mL/min/1.73m^2)	91.2 (13.6)	93.0 (13.0)	94.6 (12.4)	95.7 (12.0)	97.7 (11.5)	**<0.001**
Intraocular pressure (mmHg) [a]	15.7 (3.3)	15.9 (3.3)	16.0 (3.3)	16.0 (3.3)	16.0 (3.3)	**<0.001**
mRNFL thickness (µm) [b]	28.9 (3.9)	29.0 (3.9)	28.9 (3.8)	28.9 (3.8)	28.9 (3.8)	0.68
GCIPL thickness (µm) [c]	75.0 (5.2)	75.2 (5.2)	75.3 (5.4)	75.3 (5.2)	75.4 (5.1)	**<0.001**
Glaucoma prevalence, *n* (%) [d]	93 (1.6)	73 (1.2)	76 (1.3)	82 (1.4)	71 (1.2)	0.48
Estimated sodium intake (mg, 24-hour recall) [e]	1 775 (884)	1 863 (874)	1 943 (917)	2 000 (944)	2 035 (977)	**<0.001**

All values represent mean (standard deviation), unless otherwise specified. [a] *n* = 29 100. [b] *n* = 29 660. [c] *n* = 29 577. [d] *n* = 29 919. [e] *n* = 13 366.
HbA1c, glycated hemoglobin; MET, metabolic equivalent of task; eGFR, estimated glomerular filtration rate; mRNFL, macular retinal nerve fiber layer; GCIPL, ganglion cell-inner plexiform layer.
