# Supplementary material for: The Association of Urinary Sodium Excretion with Glaucoma and Related Traits in a Large United Kingdom Population
Source: Ophthalmol Glaucoma. Author manuscript; Available in PMC 2025 Jun 18. (PMC12174990; doi:10.1016/j.ogla.2024.04.010)
Supplement: Table S7 [file NIHMS2083578-supplement-Table_S7.pdf]

Table S7. Results of multivariable regression analyses for the association of urinary sodium excretion with glaucoma and related traits, with additional adjustment for systemic beta-blocker use and caffeine intake

Urine sodium:creatinine ratio	Intraocular pressure (mmHg) (*n* = 48 209)			mRNFL thickness (µm) (*n* = 20 461)			GCIPL thickness (µm) (*n* = 20 395)			Glaucoma prevalence (%) (*n* = 61 939)		
	**Beta**	**95% CI**	***P*-value**	**Beta**	**95% CI**	***P*-value**	**Beta**	**95% CI**	***P*-value**	**OR**	**95% CI**	***P*-value**
**Model A (without SBP)** [a]												
Continuous												
Per SD increase	0.13	0.10, 0.16	**<0.001**	-0.03	-0.08, 0.03	0.37	0.00	-0.08, 0.08	0.98	1.10	1.04, 1.16	**<0.001**
Quintiles [b]												
Quintile 1	Reference			Reference			Reference			Reference		
Quintile 2	0.14	0.04, 0.23	**0.004**	0.04	-0.12, 0.21	0.60	0.12	-0.11, 0.34	0.31	0.94	0.81, 1.09	0.40
Quintile 3	0.28	0.19, 0.38	**<0.001**	-0.08	-0.24, 0.09	0.38	0.09	-0.14, 0.32	0.43	1.01	0.87, 1.17	0.92
Quintile 4	0.34	0.24, 0.44	**<0.001**	-0.11	-0.28, 0.06	0.22	0.01	-0.22, 0.24	0.94	1.17	1.00, 1.36	**0.044**
Quintile 5	0.40	0.29, 0.50	**<0.001**	-0.07	-0.25, 0.10	0.42	0.07	-0.17, 0.31	0.59	1.26	1.08, 1.48	**0.004**
P (trend)			**<0.001**			0.14			0.93			**<0.001**
**Model B (with SBP)** [c]												
Continuous												
Per SD increase	0.08	0.05, 0.11	**<0.001**	-0.02	-0.08, 0.03	0.43	0.01	-0.06, 0.09	0.73	1.09	1.04, 1.15	**0.001**
Quintiles [b]												
Quintile 1	Reference			Reference			Reference			Reference		
Quintile 2	0.11	0.02, 0.20	**0.020**	0.05	-0.12, 0.21	0.59	0.12	-0.10, 0.34	0.29	0.94	0.81, 1.09	0.39
Quintile 3	0.23	0.13, 0.32	**<0.001**	-0.07	-0.24, 0.09	0.39	0.10	-0.12, 0.33	0.37	1.01	0.86, 1.17	0.95
Quintile 4	0.25	0.15, 0.35	**<0.001**	-0.10	-0.27, 0.07	0.25	0.03	-0.20, 0.27	0.78	1.16	1.00, 1.36	**0.050**
Quintile 5	0.26	0.16, 0.36	**<0.001**	-0.07	-0.24, 0.11	0.47	0.10	-0.14, 0.34	0.41	1.25	1.07, 1.47	**0.006**
P (trend)			**<0.001**			0.17			0.69			**<0.001**

[a] Model A adjusted for: age (years), sex (women, men), ethnicity (White, Asian, Black, Other/Mixed), Townsend deprivation index, height (cm), weight (kg), glycated hemoglobin (mmol/mol), total cholesterol (mmol/L), smoking status (never, current, former), alcohol intake (g/day), physical activity (MET-minutes/week), assessment season (Summer, Autumn, Winter, Spring), time of urine collection (morning, afternoon, evening), urinary potassium concentration (mmol/L), systemic beta-blocker use (no, yes), and caffeine intake (mg/day). [b] Details of urine sodium:creatinine ratio quintiles for each cohort are available in Table 1. [c] Model B adjusted for: as for Model A, plus systolic blood pressure (mmHg).

mRNFL, macular retinal nerve fiber layer; GCIPL, ganglion cell-inner plexiform layer; CI, confidence interval; OR, odds ratio; SD, standard deviation; SBP, systolic blood pressure.
