# Supplementary material for: The Association of Urinary Sodium Excretion with Glaucoma and Related Traits in a Large United Kingdom Population
Source: Ophthalmol Glaucoma. Author manuscript; Available in PMC 2025 Jun 18. (PMC12174990; doi:10.1016/j.ogla.2024.04.010)
Supplement: list of consortium members [file NIHMS2083578-supplement-list_of_consortium_members.pdf]

Members of the Modifiable Risk Factors for Glaucoma Collaboration

Hugues Aschard, Institute Pasteur
Mark Chia, UCL Institute of Ophthalmology
Sharon Chua, UCL Institute of Ophthalmology
Ron Do, Icahn School of Medicine at Mount Sinai
Paul Foster, UCL Institute of Ophthalmology
Jae Kang, Brigham and Women’s Hospital, Harvard Medical School
Alan Kastner, Moorfields Eye Hospital
Anthony Khawaja, UCL Institute of Ophthalmology
Jihye Kim, Harvard T.H. Chan School of Public Health
Marleen Lentjes, Örebro University
Robert Luben, UCL Institute of Ophthalmology
Kian Madjedi, UCL Institute of Ophthalmology, University of Calgary
Giovanni Montesano, Moorfields Eye Hospital
Louis Pasquale, Icahn School of Medicine at Mount Sinai
Kelsey Stuart, UCL Institute of Ophthalmology
Alasdair Warwick, UCL Institute of Cardiovascular Science
Janey Wiggs, Massachusetts Eye and Ear Infirmary, Harvard Medical School

Members of the UK Biobank Eye and Vision Consortium

Naomi Allen, University of Oxford
Tariq Aslam, The University of Manchester
Denize Atan, University of Bristol
Sarah Barman, Kingston University
Jenny Barrett, University of Leeds
Paul Bishop, The University of Manchester
Graeme Black, The University of Manchester
Tasanee Braithwaite, St Thomas' Hospital
Roxana Carare, University of Southampton
Usha Chakravarthy, Queen's University Belfast
Michelle Chan, Moorfields Eye Hospital
Sharon Chua, UCL Institute of Ophthalmology
Alexander Day, Moorfields Eye Hospital
Parul Desai, Moorfields Eye Hospital
Bal Dhillon, University of Edinburgh
Andrew Dick, University of Bristol
Alexander Doney, University of Dundee

Cathy Egan, Moorfields Eye Hospital

Sarah Ennis, University of Southampton

Paul Foster, UCL Institute of Ophthalmology

Marcus Fruttiger, UCL Institute of Ophthalmology

John Gallacher, University of Oxford

David (Ted) Garway-Heath, UCL Institute of Ophthalmology

Jane Gibson, University of Southampton

Jeremy Guggenheim, Cardiff University

Chris Hammond, King's College London

Alison Hardcastle, UCL Institute of Ophthalmology

Simon Harding, University of Liverpool

Ruth Hogg, Queen's University Belfast

Pirro Hysi, King's College London

Pearse Keane, UCL Institute of Ophthalmology

Peng Tee Khaw, UCL Institute of Ophthalmology

Anthony Khawaja, Moorfields Eye Hospital

Gerassimos Lascaratos, Moorfields Eye Hospital

Thomas Littlejohns, University of Oxford

Andrew Lotery, University of Southampton

Phil Luthert, UCL Institute of Ophthalmology

Tom MacGillivray, University of Edinburgh

Sarah Mackie, University of Leeds

Bernadette McGuinness, Queen's University Belfast

Gareth McKay, Queen's University Belfast

Martin McKibbin, Leeds Teaching Hospitals NHS Trust

Tony Moore, UCL Institute of Ophthalmology

James Morgan, Cardiff University

Eoin O'Sullivan, King's College Hospital

Richard Oram, University of Exeter

Chris Owen, St George's, University of London

Praveen Patel, Moorfields Eye Hospital

Euan Paterson, Queen's University Belfast

Tunde Peto, Queen's University Belfast

Axel Petzold, UCL Institute of Neurology

Nikolas Pontikos, UCL Institute of Ophthalmology

Jugnoo Rahi, UCL Institute of Child Health

Alicja Rudnicka, St George's, University of London

Naveed Sattar, University of Glasgow
Jay Self, University of Southampton
Panagiotis Sergouniotis, The University of Manchester
Sobha Sivaprasad, Moorfields Eye Hospital
David Steel, Newcastle University
Irene Stratton, Gloucestershire Hospitals NHS Foundation Trust
Nicholas Strouthidis, Moorfields Eye Hospital
Cathie Sudlow, University of Edinburgh
Zihan Sun, UCL Institute of Ophthalmology
Robyn Tapp, St George's, University of London
Dhanes Thomas, Moorfields Eye Hospital
Emanuele Trucco, University of Dundee
Adnan Tufail, Moorfields Eye Hospital
Ananth Viswanathan, Moorfields Eye Hospital
Veronique Vitart, University of Edinburgh
Mike Weedon, University of Exeter
Katie Williams, King's College London
Cathy Williams, University of Bristol
Jayne Woodside, Queen's University Belfast
Max Yates, University of East Anglia
Jennifer Yip, University of Cambridge
Yalin Zheng, University of Liverpool

Members of the International Glaucoma Genetics Consortium

Tin Aung, Singapore National Eye Centre
Kathryn Burdon, University of Tasmania
Li Chen, The Chinese University of Hong Kong
Ching-Yu Cheng, National University of Singapore
Jamie Craig, Flinders University
Angela Cree, University of Southampton
Victor de Vries, Erasmus Medical Centre
Sjoerd Driessen, Erasmus Medical Centre
John Fingert, University of Iowa
Paul Foster, UCL Institute of Ophthalmology
Puya Gharahkhani, QIMR Berghofer Medical Research Institute
Christopher Hammond, King's College London
Caroline Hayward, University of Edinburgh

Alex Hewitt, University of Tasmania, University of Melbourne
Pirro Hysi, King's College London
Nomdo Jansonius, University of Groningen
Fridbert Jonansson, University of Iceland
Jost Jonas, Institute of Molecular and Clinical Ophthalmology Basel
Michael Kass, Washington University
Anthony Khawaja, UCL Institute of Ophthalmology
Chiea Khor, Genome Institute of Singapore
Caroline Klaver, Erasmus Medical Centre, Radboud University Medical Centre
Jacyline Koh, Singapore National Eye Centre
Andrew Lotery, University of Southampton
Stuart MacGregor, QIMR Berghofer Medical Research Institute
David Mackey, University of Western Australia
Paul Mitchell, University of Sydney
Calvin Pang, The Chinese University of Hong Kong
Louis Pasquale, Icahn School of Medicine at Mount Sinai
Francesca Pasutto, Friedrich-Alexander-Universität Erlangen-Nürnberg
Norbert Pfeiffer, University Medical Centre Mainz
Ozren Polašek, University of Split
Wishal Ramdas, Erasmus Medical Centre
Alexander Schuster, University Medical Centre Mainz
Ayellet Segrè, Massachusetts Eye and Ear Infirmary, Harvard Medical School
Einer Stefansson, University of Iceland
Kári Stefánsson, deCODE genetics/Amgen Inc.
Gudmar Thorleifsson, deCODE genetics/Amgen Inc.
Unnur Thorsteinsdottir, deCODE genetics/Amgen Inc., University of Iceland
Cornelia van Duijn, University of Oxford
Joëlle Vergroesen, Erasmus Medical Centre
Ananth Viswanathan, UCL Institute of Ophthalmology
Veronique Vitart, University of Edinburgh
Eranga Vithana, Singapore National Eye Centre
Janey Wiggs, Massachusetts Eye and Ear Infirmary, Harvard Medical School
James Wilson, University of Edinburgh
Robert Wojciechowski, Johns Hopkins Bloomberg School of Public Health
Tien Wong, Singapore National Eye Centre
Terri Young, University of Wisconsin-Madison
